# PREVALENCE OF FATIGUE AND IMPACT ON QUALITY OF LIFE IN CASTRATION-RESISTANT PROSTATE CANCER PATIENTS: the VITAL study

**DOI:** 10.1186/s12894-019-0527-8

**Published:** 2019-10-16

**Authors:** A. Rodríguez Antolín, L. Martínez-Piñeiro, M. E. Jiménez Romero, J. B. García Ramos, D. López Bellido, J. Muñoz del Toro, A. García García-Porrero, F. Gómez Veiga

**Affiliations:** 1 Urology Department, Hospital Universitario 12 de Octubre, Madrid, Spain; 2 Urology Department, Hospital Universitario La Paz, Madrid, Spain; 3 Urology Department, Hospital Universitario de Puerto Real, Cádiz, Spain; 4 Urology Department, Complejo Hospitalario Universitario de Huelva, Huelva, Spain; 5 Urology Department, Complejo Hospitalario Universitario de Orense, Orense, Spain; 6 Janssen Medical Department, Paseo de las 12 estrellas 5-7, 28042 Madrid, Spain; 7 Urology Department, Hospital Clínico Universitario de Salamanca e Instituto de investigación Biomédica de Salamanca (GITUR-IBSAL), Salamanca, Spain

**Keywords:** Fatigue, Castration-resistant prostate cancer, Quality of life, BFI questionnaire, FACT-P questionnaire

## Abstract

**Background:**

Fatigue is one of the most prevalent symptoms among cancer patients. Specifically, in metastatic castration-resistant prostate cancer (mCRPC) patients, fatigue is the most common adverse event associated with current treatments. The purpose of this study is to describe the prevalence of fatigue and its impact on quality of life (QoL) in patients with CRPC in routine clinical practice.

**Methods:**

This was a cross-sectional, multicentre study. Male chemo-naïve adults with high-risk non-metastatic (M0) CRPC and metastatic (M1) CRPC (mCRPC) were eligible. Fatigue was measured using the Brief Fatigue Inventory (BFI) and QoL was assessed using the Functional Assessment of Cancer Therapy questionnaire for patients with prostate cancer (FACT-P) and the FACT-General (FACT-G) questionnaire. Data were analysed using Mann-Whitney or Kruskal-Wallis tests (non-parametric distribution), a T-test or an ANOVA (parametric distribution) and the Fisher or chi-squared tests (categorical variables).

**Results:**

A total of 235 eligible patients were included in the study (74 [31.5%] with M0; and 161 [68.5%] with M1). Fatigue was present in 74%, with 38.5% of patients reporting moderate-to-severe fatigue. Mean FACT-G and FACT-P overall scores were 77.6 ± 16.3 and 108.7 ± 21.4, respectively, with no differences between the CRPC M0 and CRPC M1 subgroups. Fatigue intensity was associated with decreased FACT-G/P scores, with no differences between groups. Among 151 mCRPC patients with available treatment data, those treated with abiraterone-prednisone ≥3 months showed a significant reduction in fatigue intensity (*p* = 0.043) and interference (p = 0.04) compared to those on traditional hormone therapy (HT). Patients on abiraterone-prednisone ≥3 months showed significantly better FACT-G/P scores than patients on HT (*p* = 0.046 and 0.018, respectively).

**Conclusion:**

Our data show a high prevalence and intensity of fatigue and its impact on QoL in chemo-naïve CRPC patients. There is an association between greater fatigue and less QoL, irrespective of the presence or absence of metastasis. Chemo-naïve mCRPC patients receiving more than 3 months of abiraterone acetate plus prednisone showed an improvement of fatigue and QoL when compared to those on traditional HT.

**Trial registration:**

Not applicable since it is not an interventional study.

## Background

Prostate cancer is the most frequent cancer among males in Europe [1]. In 2017, approximately 160.000 men will be diagnosed with prostate cancer adding to 3.3 million existing survivors [2].

Even though optimal disease control is achieved with androgen deprivation therapy (ADT), most patients will eventually progress and develop metastatic castration-resistant PC (mCRPC) [3], which is associated with poor prognosis.

Cancer-related fatigue is one of the most prevalent, distressing and anticipated symptoms experienced by patients across all tumours. It is not proportional to recent activity and it interferes with usual functioning [4]. In patients with mCRPC, fatigue is by far a dominant symptom of the disease and is the most common adverse event associated with treatments [5]. Manifestations include a sense of persistent physical, mental and/or emotional tiredness [6], which can cause a significant impact on quality of life (QoL) [7].

New therapeutic options for men with mCRPC have been developed over the last few years [8], including therapies targeting the androgen receptor pathway. Abiraterone acetate, a new class of anti-androgen, inhibits the synthesis of testosterone in the adrenal glands, testes and the tumour microenvironment, leading to suppression of PC growth and tumour regression [9]. In patients with mCRPC having progressed after docetaxel chemotherapy, abiraterone acetate and prednisone is the only treatment to have shown clinically meaningful improvements in fatigue [10].

Surprisingly, no studies have been conducted to evaluate the presence of fatigue in CRPC patients. The aim of this study was to describe the prevalence of fatigue and its impact on QoL in patients with both chemo-naïve mCRPC and high-risk non-metastatic CRPC in routine clinical practice.

## Methods

### Study design

The VITAL Study was a cross-sectional study, carried out in 39 specialised urological clinics across Spain between January 2015 and September 2015. The study was conducted in accordance with the Declaration of Helsinki including all amendments, and was approved by the Ethics Committee of Hospital Universitario 12 de Octubre (Madrid, Spain) as ethical reference committee. All patients gave written informed consent before their inclusion in the study, and their treatment followed routine clinical guidelines.

### Study population

Eligible patients included adult males with a histological diagnosis of high-risk non-metastatic CRPC (defined as prostate-specific antigen [PSA] doubling time [PSADT] ≤10 months; M0) or mCRPC (defined by visceral metastases, distant lymph nodes, or presence of bone metastases; M1). Patients who had participated in any investigational drug study or any expanded-access or named-patient program were excluded, as well as those who had been treated with chemotherapy previously.

### Sample size calculation

According to different studies published, fatigue is present in more than 40% of oncologic patients, increasing up to almost 90% depending on the study cohort characteristics, such as age, pathology, disease stage, etc. [11]. According to these data, an incidence of fatigue of around 65% was estimated in advanced prostate cancer patients. A total of 243 patients were needed in order to detect an incidence of fatigue of 65% with a 6% precision and a 95% confidence interval. Considering a losing rate of 5%, it was necessary to include a total of 256 patients in the study.

### Variables

Data were collected using self-report questionnaires and supplemented with clinical data from the patients’ medical records.

Fatigue was measured using the Brief Fatigue Inventory (BFI), a standard and reliable instrument used to assess fatigue in patients with cancer. The BFI is a nine-item instrument, consisting of three items assessing present, usual and worst level of fatigue and six items concerning the interference of fatigue with general activity over the previous week [12]. ‘Fatigue intensity’ was defined as the score of the worst level of fatigue in the last 24 h (BFI item 3), on a 0–10 scale, with 0 being ‘No fatigue’ and 10 being ‘As bad as you can imagine’. Fatigue was classified as mild, moderate or severe based on the score for item 3 (1–4, 5–7, or 8–10, respectively). ‘Fatigue interference’ was defined as the average score of all interference items (items 4A-4F), on a 0–10 scale, with 0 being ‘Does not interfere’ and 10 being ‘Completely interferes’. The global BFI score is the arithmetic mean of all nine items (score, 0–10). The correlation between the physicians’ and the patients’ perception of fatigue was also calculated.

QoL was assessed using the Functional Assessment of Cancer Therapy questionnaire for patients with prostate cancer (FACT-P), which has been validated to estimate QoL in men with PC [13]. This tool comprises the 27-item FACT-General (FACT-G) questionnaire, which measures QoL in cancer patients, and a 12-item prostate cancer subscale, designed to measure QoL specifically in prostate cancer. The FACT-P questionnaire is scored by adding the subscales of the FACT-G plus the prostate cancer subscale to yield a comprehensive QoL score.

Further data were recorded from the patients’ medical records and included lifestyle habits, analytical values, comorbidities, current treatment, and other factors that could be associated with fatigue (Table 3) .

### Statistical considerations

Descriptive analyses were used for the study variables.

The prevalence of fatigue was calculated as the proportion of patients suffering from fatigue (BFI item 3 score > 0), with the relevant 95% confidence interval (CI). The concordance between the patients’ and the physicians’ perception of fatigue was examined using the kappa index (κ: < 0.0 poor, 0.0–0.20 slight, 0.21–0.40 fair, 0.41–0.60 moderate, 0.61–0.80 substantial, 0.81–1.00 almost perfect) [14].

When inferential analyses were required, the Mann-Whitney or Kruskal-Wallis tests were used for variables not fitting a normal (or parametric) distribution. For variables fitting a normal (or parametric) distribution, a T-test or an ANOVA were used. In contingency tables for categorical variables, the Fisher or chi-squared tests were used. All hypothesis tests were two-sided, with a significance level of 0.05.

A logistic regression analysis was performed to evaluate the association between clinical characteristics and the presence of fatigue, based on those variables with a *p*-value < 0.2 in the bivariate analyses.

Missing data were not imputed and were left as lost. Statistical analyses were performed using the Statistical Package for the Social Sciences (SPSS) software package version 18.0.

## Results

### Patient characteristics

A total of 254 patients were included in the study. Of these, 19 subjects were excluded due to screening failures. The final evaluable population comprised 235 patients, with 74 (31.5%) in the M0 group and 161 (68.5%) in the M1 group (Table 1). At inclusion, median age for the entire patient population was 75.1 (46.2–92.4) years, median PSA value was 17.8 (6.8–43.3) ng/dL, and 90.7% of patients had an ECOG performance status grade of 0 or 1.

**Table 1:** Clinical characteristics

	M0 (*N* = 74)	M1 (*N* = 161)	Total (*N* = 235)
Age (years), median (IQR)	77.3 (71.3–81.3)	74.8 (70.0–80.4)	75.1 (70.2–80.6)
BMI (kg/m^2^), median (IQR)	28.3 (25.7–30.9)	27.7 (25.4–30.1)	27.7 (25.5–30.4)
Gleason score at diagnosis, n (%)			
≤ 7	32 (43.9)	55 (36.4)	87 (38.8)
> 7	41 (56.2)	96 (63.6)	137 (61.2)
ECOG performance status, n (%)			
ECOG 0–1	66 (89.2)	147 (91.9)	213 (91.0)
ECOG 2–3	8 (10.8)	13 (8.1)	21 (9.0)
Locally advanced and metastatic disease at diagnosis, n (%)	7 (9.6)	63 (39.9)	70 (30.3)
Extension of the disease at present, n (%) (multiple answers possible)	17 (24.3)	161 (100)	178 (75.7)
Bone	0 (0)	145 (90.1)	145 (61.7)
≤ 5 bone metastases	0	70 (48.3)	70 (29.8)
> 5 bone metastases	0	75 (51.7)	75 (31.9)
Locoregional lymph nodes	17 (23.0)	48 (29.8)	65 (27.7)
VDistant lymph nodes	0 (0)	36 (22.4)	36 (15.3)
Visceral	0 (0)	6 (3.7)	6 (2.6)
Lung	0 (0)	2 (33.3)	2 (33.3)
Liver	0 (0)	2 (33.3)	2 (33.3)
Multiple locations	0 (0)	2 (33.3)	2 (33.3)
Concomitant disease, n (%) (multiple answers possible)			
Hypertension	42 (56.8)	93 (57.8)	135 (57.4)
Diabetes mellitus	19 (25.7)	34 (21.1)	53 (22.6)
Obesity	19 (25.7)	31 (19.3)	50 (21.3)
Cardiovascular disease	17 (23.0)	32 (19.9)	49 (20.9)
Respiratory disorders	12 (16.2)	20 (12.4)	32 (13.6)
Anaemia	7 (9.5)	17 (10.6)	24 (10.2)
Depression	6 (8.1)	8 (5.0)	14 (6.0)
Laboratory parameters, median (IQR)			
PSA (ng/ml)	11.9 (7.1–30.2)	24.2 (6.6–63.8)	17.8 (6.8–43.3)
Testosterone (ng/dl)	0.3 (0.1–12)	0.3 (0.1–2.5)	0.3 (0.1–5.4)
Hb (g/dl)	13.9 (13–14.2)	13.1 (12–14)	13.4 (12.2–14.2)
LDH (UI/I)	209 (181.3–371.5)	217.5 (173.8–323.5)	216 (178.3–349.8)
Alkaline phosphatase (UI/I)	77.1 (58–104)	105 (75.8–176.8)	94 (70–147)

*BMI* Body mass index, *Hb* Haemoglobin, *IQR* Interquartile range, *LDH* Lactate dehydrogenase, *PSA* Prostate-specific antigen

### Fatigue

The prevalence of fatigue in the overall population was 74% (95% CI, 67.9–79.4%), with 38.5% (95% CI, 32.1–44.9%) of patients reporting moderate-to-severe fatigue (Table 2). The prevalence of fatigue was independent of the presence of metastases (75.3% in M0 versus 73.9% in M1, *p* = 0.817).

**Table 2:** Prevalence of fatigue

	M0 (N = 74)	M1 (N = 161)	Total (N = 235)*
No fatigue (BFI questionnaire = 0)	18 (24.7)	42 (26.1)	60 (25.6)
Mild fatigue (BFI questionnaire = 1, 2, 3 or 4)	26 (35.6)	58 (36.0)	84 (35.9)
Moderate fatigue (BFI questionnaire = 5, 6 or 7)	20 (27.4)	45 (28.0)	65 (27.8)
Severe fatigue (BFI questionnaire = 8, 9 or 10)	9 (12.3)	16 (9.9)	25 (10.7)

Missing value, n = 1

Data are expressed as n (%)

According to the physicians’ perception of fatigue, all 235 patients were classified as having fatigue (88 [37.4%]) or no fatigue (147 [62.6%]). Regarding the patients’ self-perception, there were 86 (36.6%) subjects with fatigue and 149 (63.4%) with no fatigue. When these two approaches were compared, 77 (32.8%) subjects were classified as having fatigue and 138 (58.7%) as no fatigue. Overall these two approaches showed an “almost perfect” concordance, with κ = 0.818.

The bivariate and multivariate analyses revealed that respiratory and cardiovascular disorders were the only factors significantly associated with the presence of fatigue based on the response to the BFI item 3 score > 0 (odds ratio [OR] 4.7 and 3.6, respectively; Table 3).

**Table 3:** Factors associated with fatigue

	Bivariate		Multivariate	
	OR (95% CI)	*p-value*	OR (95% CI)	*p-value*
Age	1.032 (0.995–1.071)	0.089	–	–
PSA	1.001 (0.999–1.003)	0.464	–	–
Testosterone	0.984 (0.956–1.012)	0.248	–	–
Hb	0.821 (0.666–1.012)	0.065	–	–
LDH	0.999 (0.996–1.003)	0.680	–	–
ALP	1.001 (0.998–1.005)	0.503	–	–
Albumin	0.404 (0.159–1.025)	0.056	–	
Sodium	0.889 (0.782–1.010)	0.071	–	–
Alcohol consumption	2.750 (0.793–9.542)	0.111	–	–
Regular exercise	0.550 (0.294–1.031)	0.062	–	
Diabetes mellitus	1.918 (0.873–4.212)	0.105	–	–
Cardiovascular disorders	4.884 (1.676–14.233)	0.004	4.7 (1.6–13.9)	0.005
Respiratory disorders	3.800 (1.113–12.969)	0.033	3.6 (1.0–12.5)	0.043
High blood pressure	1.522 (0.843–2.746)	0.163	–	–
Sleep disturbances	3.982 (0.503–31.511)	0.191	–	–
Time since diagnosis	1.054 (0.981–1.132)	0.150	–	
Surgical castration	0.246 (0.53–1.131)	0.072	–	–
Chemical castration	0.000 (0.000-)	1.000	–	–
Radical prostatectomy at diagnosis	0.578 (0.273–1.225)	0.153	–	–
External radiation therapy at diagnosis	1.747 (0.792–3.851)	0.167	–	–
Duration of previous LHRH therapy		0.757		
7–12 months	1.000 (0.084–11.931)	1.000	–	–
13–18 months	0.583 (0.052–6.587)	0.663	–	–
19–24 months	1.111 (0.097–12.750)	0.933	–	–
25–35 months	0.905 (0.080–10.210)	0.935	–	–
36–47 months	0.857 (0.076–9.695)	0.901	–	–
48–59 months	0.571 (0.049–6.606)	0.654	–	–
≥ 60 months	1.333 (0.131–13.586)	0.808	–	–
Urinary catheter	3.857 (0.527–28.241)	0.184	–	–
Haematuria	4.370 (0.556–34.346)	0.161	–	–

*CI* Confidence interval, *Hb* Haemoglobin, *LDH* Lactate dehydrogenase, *LHRH* Luteinizing hormone-releasing hormone, *OR* Odds ratio, *PSA* Prostate-specific antigen

### QoL outcomes

**Fig. 1:**
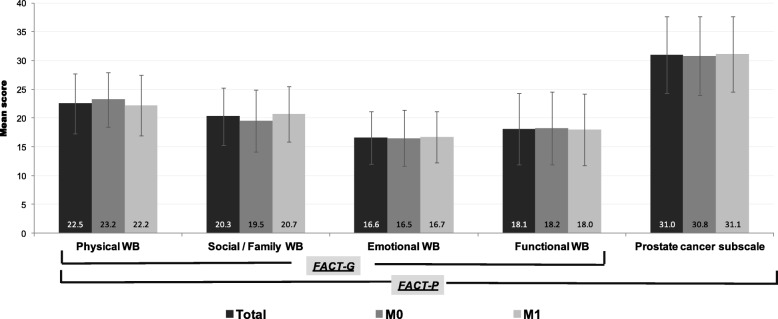
Results of the FACT-G and FACT-P questionnaires per study groups

Mean FACT-G and FACT-P overall scores were 77.6 ± 16.3 and 108.7 ± 21.4, respectively. We compared M0 to M1 for their overall score on the QoL questionnaires, finding that both groups showed similar levels of functional status. The mean FACT-G score was 77.5 ± 17.0 for M0 versus 77.6 ± 16.0 for M1 (*p* = 0.955) and the mean FACT-P score was 108.6 ± 21.7 for M0 versus 108.7 ± 21.3 for M1 (*p* = 0.966). The mean scores for the domains of the FACT-G and FACT-P scales per study groups are displayed in Fig. 1.

An association with fatigue intensity was seen across all QoL measures. Patients who reported greater fatigue intensity showed lower QoL, with worse mean FACT-G and FACT-P scores. This association was found to be independent of absence or presence of metastases (Table 4).

**Table 4:** Interference of fatigue with QoL instruments in the study cohorts

	FACT-G			FACT-P		
	M0	M1	Total	M0	M1	Total
No fatigue	91.1 ± 11.8	86.7 ± 13.8	88.0 ± 13.3	127.6 ± 16.9	122.1 ± 16.8	123.7 ± 16.9
Mild fatigue	80.7 ± 12.5	78.6 ± 13.2	79.3 ± 12.9	111.1 ± 14.8	109.6 ± 17.0	110.1 ± 16.3
Moderate fatigue	67.5 ± 13.8	73.3 ± 14.9	71.5 ± 14.7	96.5 ± 15.9	102.8 ± 20.5	100.8 ± 19.3
Severe fatigue	65.7 ± 21.8	61.8 ± 18.1	63.2 ± 19.2	90.5 ± 27.7	86.2 ± 24.3	87.7 ± 25.1
*p*-value	< 0.001	< 0.001	< 0.001	< 0.001	< 0.001	< 0.001

Data are expressed as mean ± SD

### Fatigue and QoL in mCRPC according to treatment

Among all 161 mCRPC patients, 151 had available treatment data: 75 (50%) patients were receiving traditional hormone therapy (HT; mostly bicalutamide and flutamide given that during the recruitment of this study, no new anti-androgen drug such as apalutamide or enzalutamide was commercially available) and 76 (50%) were on abiraterone-prednisone. These were in turn classified based on treatment duration: 33 (22%) patients had been receiving treatment for < 3 months and 43 (28%) for ≥3 months. Table 5 shows the comparison of fatigue and QoL outcomes across these three cohorts. Patients receiving abiraterone acetate plus prednisone ≥3 months showed a significant reduction in median fatigue intensity (2.0 [0.0–9.0] versus 3.0 [0.0–10.0]; *p* = 0.043) and median fatigue interference (1.8 [0.0–10.0] versus 2.7 [0.0–9.0]; p = 0.04) as compared to those on HT. The proportion of patients with clinically significant fatigue (BFI item 3 score ≥ 5) was lower among patients with ≥3 months of abiraterone acetate plus prednisone treatment than in those receiving HT (25.6% versus 41.3%), although this difference did not reach statistical significance. Significantly better median FACT-G and FACT-P scores were found in patients treated with abiraterone acetate plus prednisone for ≥3 months in comparison to patients treated with HT (FACT-G: 77.0 [24.7–101.0] versus 83.0 [38.0–103.0], *p* = 0.046; FACT-P: 108.3 [50.7–140.0] versus 117.0 [61.0–138.0], *p* = 0.018).

**Table 5:** Comparison of Fatigue and QoL outcomes in mCRPC patients

	HT (*n* = 75)	AAP < 3 months (*n* = 33)	AAP ≥ 3 months (*n* = 43)	*p*-value	*p-value* AAP ≥ 3 months vs AAP < 3 months	*p-value* AAP ≥ 3 months vs HT
Fatigue intensity (BFI item 3)	3.0 (0.0–10.0)	4.0 (0.0–8.0)	2.0 (0.0–9.0)	0.125^a^	0.243	0.043
Fatigue severity, n (%)				0.039	–	–
*No fatigue*	15 (20.0)	10 (30.3)	13 (30.2)			
*Mild fatigue*	29 (38.7)	8 (24.2)	19 (44.2)			
*Moderate fatigue*	18 (24.0)	14 (42.4)	9 (20.9)			
*Severe fatigue*	13 (17.3)	1 (3.0)	2 (4.7)			
Interference (BFI item 4A-4F)	2.7 (0.0–9.0)	2.7 (0.0–7.0)	1.8 (0.0–10.0)	0.106^b^	0.473	0.04
FACT-G overall score	77.0 (24.7–101.0)	81.0 (35.0–101.0)	83.0 (38.0–103.0)	0.121^c^	0.429	0.046
FACT-P overall score	108.3 (50.7–140.0)	114.3 (57.0–137.0)	117.0 (61.0–138.0)	0.052^d^	0.402	0.018
*Physical WB*	22.0 (4.0–28.0)	24.0 (12.0–28.0)	24.0 (9.0–28.0)	0.031^e^	0.643	0.014
*Social / Family WB*	21.0 (3.0–28.0)	21.0 (2.0–28.0)	20.0 (11.0–28.0)	0.834	–	–
*Emotional WB*	17.0 (3.0–24.0)	17.0 (7.0–23.0)	18.0 (7.0–23.0)	0.727	–	–
*Functional WB*	17.0 (0.0–28.0)	19.0 (3.0–27.0)	21.0 (6.0–28.0)	0.041^f^	0.054	0.017
*Prostate cancer subscale*	30.0 (11.0–42.0)	32.0 (17.0–43.0)	34.0 (20.0–43.0)	0.029^g^	0.462	0.008

Data are expressed as median (IQR), unless otherwise stated

*AAP* Abiraterone Acetate-prednisone, *HT* traditional hormone therapy, *IQR* interquartile range, *WB* well-being

## Discussion

To the best of our knowledge, this is the first observational study in the setting of routine clinical practice that specifically evaluates self-reported fatigue and its impact on QoL in chemo-naïve patients with CRPC, using well-established validated instruments for this purpose.

Besides pain, fatigue is the most distressing and predominant symptom reported by patients with mCRPC [15]. We found that almost three quarters of our study population were suffering from fatigue, regardless of the presence of metastases, and a high proportion of patients were suffering from moderate-to-severe fatigue. The prevalence of fatigue has been studied previously, ranging from 39 to 90% [11]; however, the prevalence rates for cancer-related fatigue vary widely depending on how fatigue is defined and assessed.

Even though cancer-related fatigue has a terrible impact on daily activities and is one of the main drivers of poor QoL [16], a poor correlation has long been observed between clinician-perceived and patient-reported subjective symptoms, such as fatigue [17–19]. Surprisingly, in our study we observed an improvement in the level of agreement between the clinicians’ and the patients’ perception of fatigue, finding an excellent concordance between the two. This highlights the importance of the need for assessing fatigue symptoms on an ongoing basis and developing management plans to increase health-care provider awareness of early fatigue symptoms, in order to help patients and their primary carers to recognise fatigue symptoms early, and thereby increase QoL in this group of patients.

A list of possible correlates of fatigue in mCRPC was proposed recently by Colloca et al., grouping them in cancer-related (anemia, pain, etc), patient-related (physical function, liver dysfunction, etc) and treatment-related (hormonal therapy, chemotherapy, etc) [5]. The logistic regression model developed in this study has revealed that beyond initial therapy or biological parameters, “patient-related” respiratory and cardiovascular disorders were the most important explanatory factors associated with fatigue. In bivariate analysis, hemoglobin and the practice of regular exercise seem to have some value but did not reach statistical significance. Interestingly, the time in treatment with analogues had no impact on fatigue in our study.

In this study, we found that PC patients showed similar levels of functionality, as measured by the FACT-P questionnaire, irrespective of the absence or presence of metastases. In light of previous studies, this was a rather unexpected finding, as the prevalence of cancer-related fatigue is likely to increase as the disease progresses [10, 20]. We observed that fatigue intensity was directly related to impaired QoL across all dimensions of the FACT-G and FACT-P instruments. This is in line with previous studies, in which fatigue was the most common symptom and the most significant predictor of impaired QoL [21]. As a multidimensional symptom, fatigue can affect specific dimensions of the QoL instruments, for which measurement of intensity alone is rather inappropriate. The findings reported by Gupta et al. [22] have essential implications in clinical practice. The authors highlighted that patients with PC at close monitoring of QoL, coupled with an improvement in fatigue, dyspnea and cognitive function within 3 months of treatment, were at a significantly decreased risk of mortality.

Our findings are of practical importance to mCRPC treatment and further support abiraterone as a valuable option for the treatment of mCRPC patients. Sternberg et al. [10] reported the results of the first phase III clinical trial in the setting of advanced prostate cancer to specifically evaluate patient-reported fatigue outcomes, highlighting that abiraterone-prednisone was associated with improvements not only in fatigue intensity but also in fatigue interference, and that this was perceivable and meaningful to patients. The AQUARiUS study [23] also added evidence supporting the benefits of abiraterone-prednisone treatment regarding fatigue. In this observational study, fatigue and cognition was evaluated in mCRPC patients receiving either abiraterone-prednisone or enzalutamide. Abiraterone-prednisone showed favourable effect on fatigue across all fatigue scales evaluated, proving significant difference at 3 months of treatment comparing to enzalutamide. In keeping with these, we have found that chemo-naïve mCRPC patients receiving more than 3 months of treatment with abiraterone-prednisone had lower levels of fatigue and better QoL compared to traditional hormone therapy, which could not be ascribed to differences in previous chemotherapy exposure. Despite all these findings, we cannot determine the mechanism underlying the benefits associated with a longer duration of treatment with abiraterone-prednisone, which could be the result of amelioration of disease progression. Nonetheless, these findings should guide new longitudinal studies to confirm the results.

The cross-sectional design is probably the most important limitation of our study. In common with all cross-sectional studies, we can only offer a ‘snapshot’ of the current situation. It may have been better to follow the patients throughout a longer period of time, but this would have taken much longer, and we probably would have needed to increase the sample size. It should also be noted that, as an observational study design, certain biases might have been introduced when collecting the data. These might affect the interpretation of the results. However, conducting this type of studies –in real life– is of great relevance, as they help us learn about the conditions derived from routine clinical practice.

## Conclusions

Our data show high prevalence rates and high intensity of fatigue with a significant impact on QoL in high-risk M0 CRPC and chemo-naïve mCRPC patients. There is an association between more fatigue and less QoL, which is independent of the presence or absence of metastases. Finally, chemo-naïve mCRPC patients receiving more than 3 months of abiraterone-prednisone showed an improvement of fatigue and QoL compared to patients on traditional HT.

## Supplementary information

**Additional file 1.** Participant Sites VITAL STUDY

## Data Availability

The datasets used and/or analysed during the current study are available from the corresponding author on reasonable request.

## Abbreviations

ADT: Androgen Deprivation Therapy
BFI: Brief Fatigue Inventory
CRPC: Castration Resistant Prostate Cancer
FACT-G: FACT-General
FACT-P: Functional Assessment of Cancer Therapy questionnaire for patients with prostate cancer
HT: Hormone Therapy
M0: Non-metastatic
M1: Metastatic
mCRPC: Metastatic Castration Resistant Prostate Cancer
PC: Prostate Cancer
PSA: Prostate Specific Antigen
PSADT: Prostate Specific Antigen Doubling Time
QoL: Quality of Life
