# Supplementary material for: PREVALENCE OF FATIGUE AND IMPACT ON QUALITY OF LIFE IN CASTRATION-RESISTANT PROSTATE CANCER PATIENTS: the VITAL study
Source: BMC Urol. 2019 Oct 16;19:92. doi: 10.1186/s12894-019-0527-8 (PMC6796370; doi:10.1186/s12894-019-0527-8)
Supplement: Supplementary file 1 — Additional file 1. Participant Sites VITAL STUDY [file 12894_2019_527_MOESM1_ESM.docx]

**Participant Sites VITAL STUDY**

| **Site** | **Number** |
| --- | --- |
| H. Infanta Sofía | 001 |
| H. San Juan de Alicante | 002 |
| H. Elche | 003 |
| H. La Princesa | 004 |
| H. Mar | 005 |
| H. Ramón y Cajal | 006 |
| H. Infanta Cristina | 007 |
| H. Clínico de Valencia | 008 |
| H. Josep Trueta | 009 |
| H. Orense | 010 |
| H. Son Espases | 011 |
| H. Manacor | 012 |
| H. Salamanca | 013 |
| H. Mérida | 014 |
| H. Santiago | 015 |
| H. Lozano Blesa | 016 |
| H. La Fe | 017 |
| H. Juan Ramón Jiménez | 018 |
| H. San Agustín | 019 |
| H. Valme | 020 |
| H. Gregorio Marañón | 021 |
| H. Puerto Real | 022 |
| H. Costa del Sol | 023 |
| H. 12 de Octubre | 024 |
| H. Virgen del Camino | 025 |
| H. Carlos Haya | 026 |
| H. Parc Taulí | 027 |
| H. La Ribera | 028 |
| H. Bellvitge | 029 |
| H. Jaén | 030 |
| H. Ferrol | 031 |
| Fundación Puigvert | 032 |
| H. Santa Lucía | 033 |
| H. Vall d´hebron | 034 |
| H. Virgen de la Luz | 035 |
| H. Rey Juan Carlos | 036 |
| Fundación Jiménez Díaz | 037 |
| H. Univ. de Canarias | 038 |
| H. Ciudad Real | 039 |
